# Hepaticojejunostomy Anastomosis Worm Obstruction and Its Laparoscopic Management: A Case Report and Review of Literature

**DOI:** 10.7759/cureus.21968

**Published:** 2022-02-07

**Authors:** Jayanta Kumar Das, Gordon M Rangad

**Affiliations:** 1 Department of General and Minimally Invasive Surgery, Nazareth Hospital, Shillong, IND

**Keywords:** ureterorenoscope, enteroscopy, sepsis, cholangitis, biliary ascariasis, roux-en-y hepaticojejunostomy

## Abstract

We report a surprising case of intraoperatively detected worm obstruction of a hepaticojejunostomy anastomosis. The patient presented with acute cholangitis including fever, abdominal pain, obstructive jaundice and sepsis. Six years earlier, she had undergone open cholecystectomy with a right subcostal incision. Ultrasonography that night depicted the absence of the gall bladder and the presence of apparent stones in the common hepatic and common bile ducts. The patient was posted for laparoscopic exploration of common bile duct. Intraoperatively, worm obstruction was found in the hepaticojejunostomy anastomosis created during the previous operation. The obstruction was managed laparoscopically, and the patient recovered without any complications and was monitored for two years. In a search of PubMed and Google Scholar, we found reports of laparoscopy-assisted endoscopic retrograde cholangiopancreatography as an established method of relieving hepaticojejunostomy obstruction; however, we found no case of laparoscopic extraction of obstructing worms. Laparoscopic exploration of a hepaticojejunostomy anastomosis through the afferent Roux loop is a feasible and safe alternative to other advanced methods of endoscopic retrograde cholangiopancreatography, for which special technique, logistics, and training are required but may not be available in many parts of the world.

## Introduction

Ascariasis is an endemic parasitic infestation in developing countries with precarious socioeconomic, health, and sanitary conditions [1-3]. It is most common in tropical and subtropical countries because of the warm and humid conditions of the soil [4]. It affects mainly children between 5 and 15 years of age [5]. Adult worms live in the small intestine, mostly in the jejunum [2,6]. These parasites have a propensity to explore orifices, ducts, and cavities [2,3,6-7]. They can migrate to bizarre places such as the biliary tree, pancreatic ducts, lungs, appendix, Meckel’s diverticulum, and peritoneal cavity [2,4-7]. Paul mentioned many reported cases of the presence of Ascaris worms in very unusual sites such as the lacrimal sac, external auditory meatus, urinary bladder, larynx, intrapulmonary bronchus, right ventricle of the heart, and pulmonary arteries [7]. From the duodenum, the worms may enter the ampulla and advance into the biliary tree. Migration of the worms into the biliary ducts is often noticed after cholecystectomy, choledochostomy, sphincterotomy, or sphincteroplasty [4]. After cholelithiasis, it is the second most common cause of acute biliary symptoms worldwide [4].

In the biliary tree, residual dead worms may cause destruction of the biliary tree epithelium, which results in fibrosis [5]. Fragments of dead worms or ova may serve as niduses for stone formation or may cause cholangitis and liver abscesses [6]. Live worms may repeatedly migrate into and out of the orifice of the ampulla of Vater [6].

If the worms are located in the intrahepatic bile ducts, anthelmintics such as mebendazole have little effect because they are poorly absorbed and do not reach therapeutic levels in the biliary tract [2,5]. Although conservative treatment is the mainstay in such cases, some form of intervention is necessary to eliminate these parasites, especially in patients with complications of the infestation [1,6].

In a patient with altered bilioenteric anatomy, routine endoscopic access to the biliary tree is difficult or impossible [8-9]. With increasing number of obesity surgeries, performing a conventional endoscopic retrograde cholangiopancreatography (ERCP) in patients with Roux-en-Y gastric bypass (RYGB) can be extremely challenging because of the long Roux limb and multiple luminal angulations and looping [8-10]. Management of biliary duct complications in such patients is still an endoscopic or surgical challenge [11-13]. Various options for patients with biliary stricture or obstruction by calculi include percutaneous transhepatic biliary intervention, peroral single-balloon enteroscopy or double-balloon enteroscopy (DBE), short-type DBE, spiral enteroscopy, percutaneous transjejunal biliary intervention (PTJBI), laparoscopy-assisted ERCP (including transgastric or transjejunal ERCP), and, as a last resort, conventional surgery [8,11-12,14-16].

## Case presentation

One evening, a 27-year-old woman presented with symptoms of acute cholangitis, including severe abdominal pain, fever, and jaundice with sepsis. She had a high total leukocyte count, abnormal results of kidney function tests and liver function tests, and a high procalcitonin level (Table 1).

**Table 1:** Some important investigations conducted on admission of the patient.

Blood tests done	Results
Total leucocyte count	25,590/cubic millimeter
Differential leucocyte count	Neutrophil 75%, lymphocytes 14%, monocytes 3%, metamyelocytes 2%, band form 6%
Serum urea	97 milligram/deciliter
Serum creatinine	0.8 milligram/deciliter
Serum total bilirubin	7.0 milligram/deciliter
Unconjugated bilirubin	0.7 milligram/deciliter
Conjugated bilirubin	6.3 milligram/deciliter
Serum alkaline phosphatase	217 unit/liter
Procalcitonin	89.53 nanogram/milliliter

The patient had undergone open cholecystectomy approximately six years earlier in another hospital. Urgent abdominal ultrasonography was performed by a novice sonologist that night; the images showed mildly dilated intrahepatic biliary radicles with an 18-mm stone in the common bile duct (CBD) and a 27-mm stone in the common hepatic duct. The gall bladder was not seen. She was admitted to the intensive care unit, and resuscitation was performed overnight. Because our institution does not have the facilities for ERCP/ magnetic resonance cholangiopancreatography (MRCP), we posted her for laparoscopic exploration of the CBD without any further investigations the next day.

**Figure 1:**
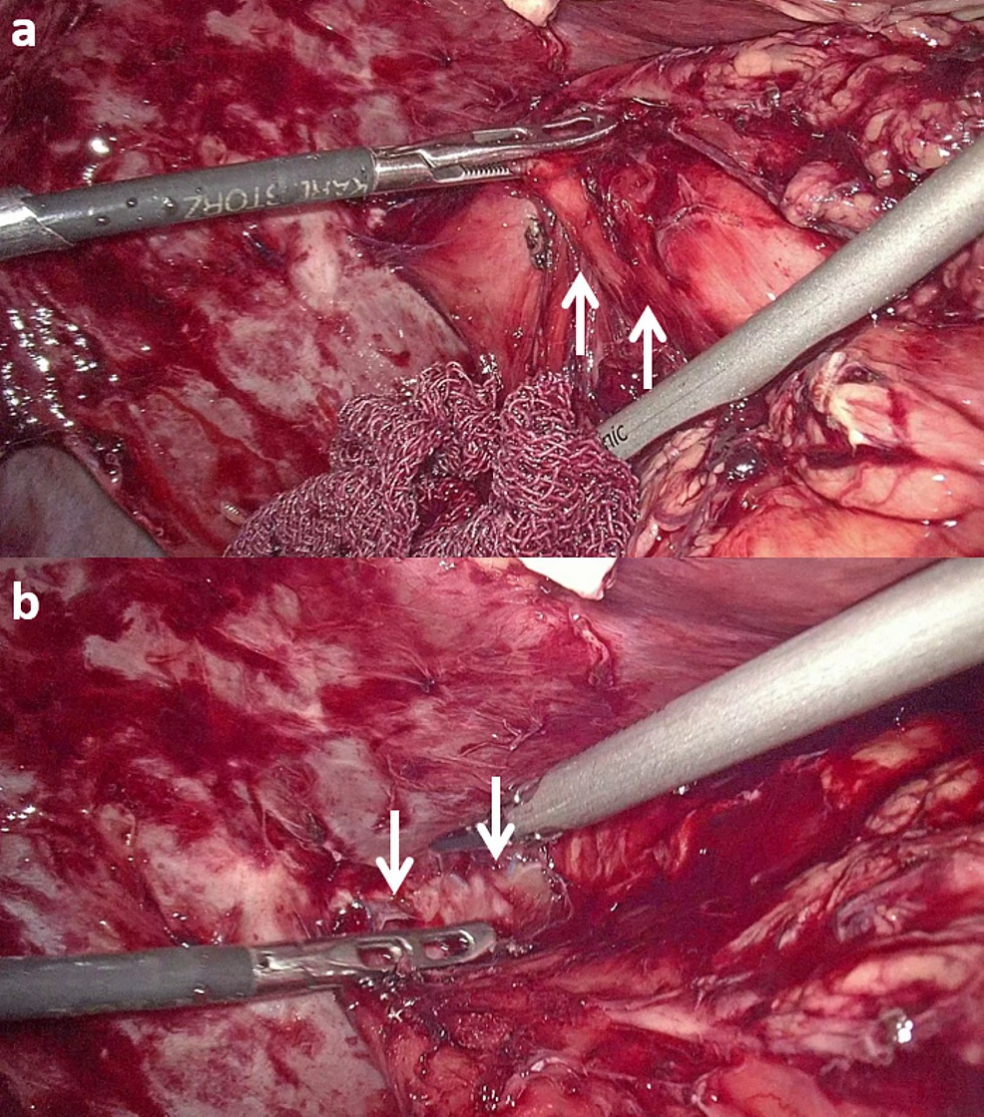
(a) Roux loop of jejunum (white arrows) reaching the liver. (b) Hepaticojejunostomy anastomosis suture line (white arrows).

**Figure 2:**
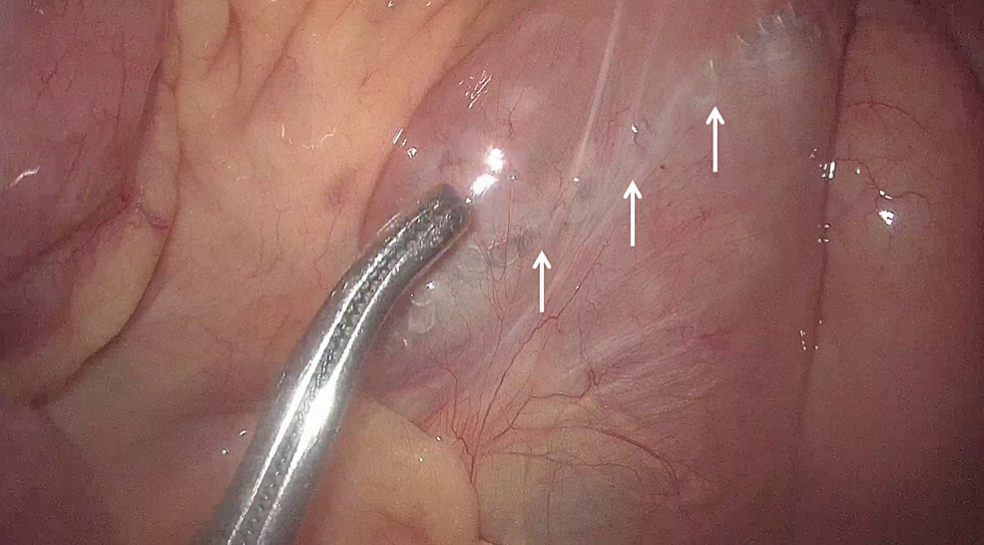
Stapled jejunojejunostomy anastomosis (white arrows).

**Figure 3:**
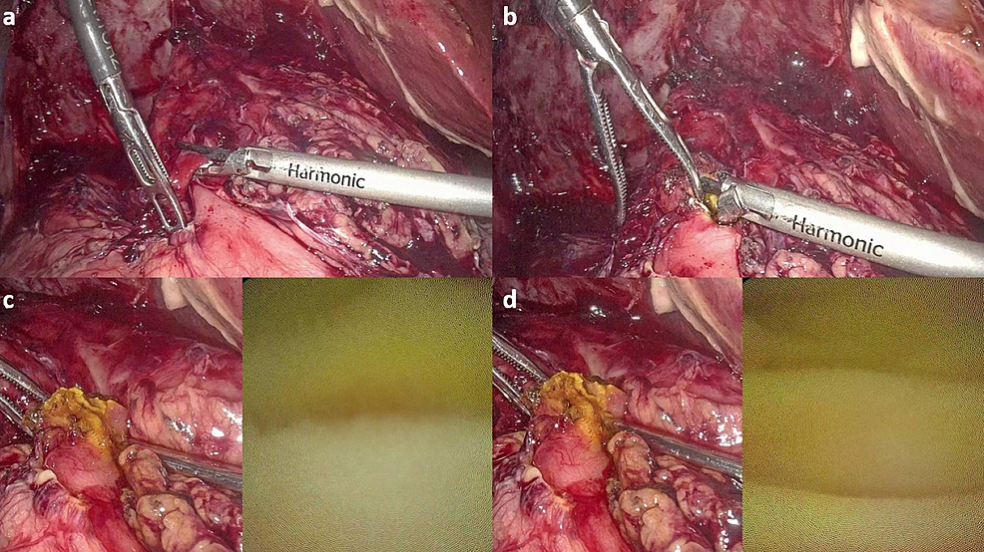
(a, b) Jejunotomy made over the Roux loop of jejunum. (c, d) Roux loop explored with an ureterorenoscope. One can see the jejunal mucosa on the right side of the pictures.

**Figure 4:**
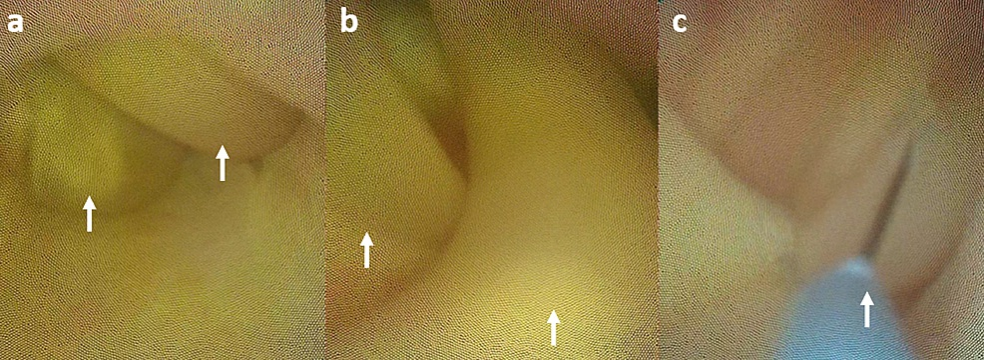
(a) Worms (white arrows) resembling stones at the site of hepaticojejunostomy. (b) Worms (white arrows) could be seen and identified properly. (c) Worms (white arrow) being basketed out.

To our surprise, we found that the patient had undergone cholecystectomy and Roux-en-Y hepaticojejunostomy (RYHJ) during the previous surgery. By gentle dissection and adhesiolysis, we identified the Roux loop, the hepaticojejunostomy (HJ) anastomosis (Figure 1), and the jejunojejunal anastomosis (Figure 2). We decided to explore the HJ anastomosis through the Roux loop of the jejunum. We made a small enterotomy in the Roux loop a few centimeters proximal to the anastomosis and explored the loop and the bile duct with the help of a semirigid ureterorenoscope (Figure 3), which we use routinely for laparoscopic exploration of the CBD. The next surprising finding was that the anastomotic area was blocked by a ball of live and dead worms (Figure 4). All the worms were successfully basketed out.

**Figure 5:**
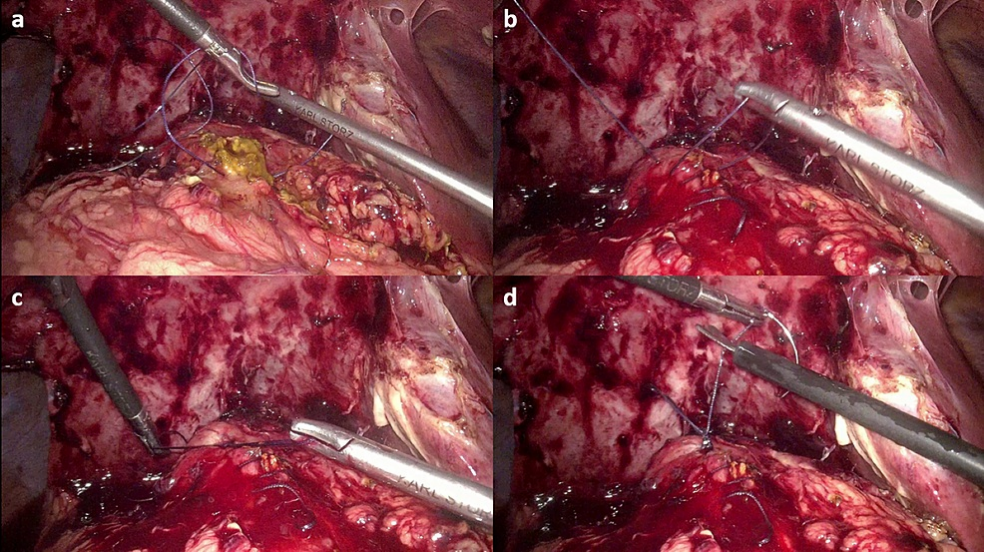
Jejunotomy closure with continuous 2-0 polyglactin sutures.

The intrahepatic ducts were washed thoroughly with normal saline to remove the collected sludge and infected bile. The jejunotomy was closed with single-layer 2-0 polyglactin continuous sutures (Figure 5). Because the efferent Y loop and jejunojejunal anastomosis were not disturbed, the patient was allowed oral intake 8 hours after the procedure, initially with liquids. She was discharged on the sixth postoperative day, after one week of intravenous antibiotics. She was given albendazole for deworming before discharge and twice a year afterward. She was in good condition at the two-year follow-up.

Literature review

Using the key words “roundworm obstruction of hepaticojejunostomy anastomosis,” we searched PubMed for literature related to our case, but we found no such literature; then we searched with “biliary ascariasis after Roux-en-Y hepaticojejunostomy” and found only one available publication. A search with “biliary ascariasis after hepaticojejunostomy” yielded two more publications. Another search with "bilioenteric anastomosis obstruction by ascariasis" yielded one more publication. After examining the references of these publications, we retrieved another case report from PubMed.

Similarly, we searched Google Scholar with the same key words. We examined the title and abstracts of the 161 publications we found, but only two were relevant to our topic; however, these had already been found during the PubMed search.

All the identified publications were case reports. Therefore, to the best of our knowledge, a total of six cases of biliary ascariasis after abdominal surgery, including ours, have been reported (Table 2). We then reviewed various approaches to biliary tree deworming in patients with altered bilioenteric anatomy.

**Table 2:** Available case reports found on literature search, including the present case. HJ, hepaticojejunostomy; RYHJ, Roux-en-Y hepaticojejunostomy

Serial number	Author and year of publication	Age/sex	Operation performed previously	Intervention performed	Gap from initial operation
1	Kasat et al., 1998 [17]	7/F	Roux-en-y choledochojejunostomy for type I choledochal cyst	Re-exploration, opening up the anterior layer of anastomosis, removal of worm, and resuturing of anastomosis	8 days
2	Braga et al., 2000 [5]	6/F	HJ for choledochal cyst	Laparotomy and removal of worm through Roux loop	3 months
3	Mercado et al., 2006 [18]	62/F	HJ for bile duct injury during laparoscopic cholecystectomy	laparotomy, removal of worm and stones, and revision HJ	7 years
4	Valentim et al., 2009 [3]	48/F	RYHJ and subcutaneous fixation of end of the Roux loop	Access to the subcutaneous jejunal loop, endoscopy, and removal of stones and worm (under local anesthesia)	11 years
5	Heimes et al., 2013 [1]	28/F	HJ for complicated cholecystectomy and pancreatic cyst excision	Laparotomy, removal of worm and stones, and revision HJ	11 years
6	Present case	27/F	RYHJ for biliary stones	Laparoscopic jejunotomy, exploration of anastomosis, and removal of worms	6 years

## Discussion

The development of bile duct stones is an adverse postoperative event in patients with a hepatobiliary-pancreatic disease who undergo HJ [14]. Such patients may also have bile duct worms, especially in endemic areas, as represented by our patient. Although plenty of literature on intestinal or extraintestinal ascariasis, including biliary ascariasis, is available, biliary ascariasis after bilioenteric bypass has rarely been reported.

Abdominal ultrasonography is inexpensive, easily available, quick, safe, noninvasive, and highly accurate for the diagnosis of biliary ascariasis [4]. It can be repeated multiple times without any adverse effects. We relied on the initial ultrasound finding of stones in the CBD and common hepatic duct in our patient and patient was posted for laparoscopic exploration of the CBD because the patient was septic.

In patients with altered anatomy as a result of bilioenteric anastomosis, traditional ERCP or endoscopic access to the biliary tree may be extremely challenging or impossible [8-10]. The main obstacles are the instrumentation of the anastomosis, length of the afferent limb (distance needed by the endoscope to traverse), excess looping, cannulation of the papilla, and lack of customized endoscopes and accessories [8-10]. Fontein et al. mentioned that for most affected patients, a single intervention, either surgical or endoscopic, may be sufficient, but a single approach may be difficult in patients who have recurrent biliary strictures or intrahepatic stones [15].

Percutaneous transhepatic cholangiography (PTC) is a well-described method in such patients. It can precisely localize the affected bile duct and allows repeated interventions through the percutaneous tract. However, preparation for safe and effective PTC may require days to weeks, and access to multiple bile ducts may not be achieved in a single puncture [11,14].

PTJBI was first described by Fang and Chou and Hutson et al. [19]. In comparison with PTC, it has the advantage of enabling repeated biliary interventions without repeated hepatic catheterizations. In 1998, McPherson et al. reported promising results after PTJBI [19]. In a review of 63 patients who had undergone PTJBI, Fontein et al. concluded it to be very safe and effective in the management of biliary strictures or calculi, with a high success rate and short hospitalization [15]. Valentim et al. reported the case of a patient with cholangitis who had undergone RYHJ 11 years earlier for Grumbach-Auvert disease, during which the end of the Roux loop was fixed subcutaneously to facilitate access to the anastomosis. With the patient under local anesthesia, they could access the jejunum and performed endoscopy through an enterotomy. A few small stones and a worm were removed from the bile duct [3].

Over the past two decades, numerous cases of laparoscopy-assisted ERCP, via either transgastric or transjejunal approaches, have been reported by various authors [8-9,11-13,20]. Dalmonte et al. stated that transjejunal ERCP is preferable to transgastric ERCP because it can be performed even when the gastric remnant is not present [20]. They warned against intra-abdominal access to the bowel because of the risk of unnecessary contamination. A few authors have mentioned that bringing out the jejunal loop by a mini-laparotomy prevents peritoneal contamination [8,11-12,20]. However, Baca-Arzaga et al. mentioned that a mini-laparotomy could result in morbidity after the surgery [11]. Moreover, in obese patients, the laparotomy wound might need to be large. In our patient, we removed the obstructing worms through a jejunotomy made in the Roux loop. Instead, of bringing out the jejunal loop to the surface, we introduced the ureterorenoscope through a small jejunotomy and finished the procedure laparoscopically. Our patient recovered without any complications.

In a meta-analysis, Ayoub et al. concluded that laparoscopy-assisted ERCP is significantly more effective than enteroscopy-assisted ERCP in patients with RYGB, but the rate of adverse effects is higher and the procedure is longer [16]. Surdeanu et al. mentioned that in patients with gastric bypass who need ERCP, the transgastric approach is usually preferred because the stomach wall is thicker than the bowel wall; therefore, the stomach is able to better withstand the forces exerted by the ureterorenoscope, and a stricture or an obstruction is less likely to form [13].

Newer endoscopic approaches such as single-balloon enteroscopy, DBE, and endoscopic abdominal ultrasonography-guided access to the biliary tree are promising. However, their availability and the necessary expertise may be limited, especially in resource-limited areas such as Shillong, India. In a comparison of ERCP via gastrostomy and DBE in patients with prior RYGB, Choi et al. concluded that the ERCP is more effective but is hindered by the delay in maturation of the gastrostomy tract, and the rate of morbidity is high [10]. They stated that DBE is a feasible and less invasive approach but has multiple limitations. In one of the first comparisons of PTC and short-type DBE in the management of bile duct stones after HJ, Tsutsumi et al. concluded that short-type DBE was useful, with low rates of adverse events and short hospitalization, and more cost-effective [14].

Kasat et al. reported a case of bile leak from a Roux-en-Y choledochoduodenostomy starting on the fifth postoperative day, caused by worms. They performed reexploration on the eighth postoperative day and found no frank leak, but a dead worm was completely blocking the lumen in the jejunum at the porta hepatis. The anterior wall of the anastomosis was opened, the worm was removed, and the anastomosis was repaired [17]. Braga et al. reported a case of roundworm obstruction of the site of HJ with resulting cholangitis and hepatic microabscesses. They had to perform laparotomy and remove the worm through a jejunotomy in the Roux loop [5]. Similarly, Heimes et al. reported the case of a woman who had previously undergone RYHJ and presented with cholangitis; MRCP suggested choledocholithiasis with cholangitis and biliary stricture. After failed ERCP, she underwent PTC, which clearly depicted the previous RYHJ but failed to resolve the problem. She underwent revision HJ for severe stricture of the anastomosis, during which multiple stones and a worm were extracted [1]. Similarly, Mercado et al. reported a case of HJ malfunction caused by worms and stones, which they operated and performed a new HJ [18]. This suggests that open surgical management may still be the last resort or the only option when other methods fail or are not available.

The literature indicates that women are more commonly affected by biliary ascariasis than are men [4]. In all the reported cases, including ours, the patients were women. However, this female predominance in the reported cases may be only coincidental.

## Conclusions

Obstruction of the biliary tract by worms after a bilioenteric bypass has rarely been reported. Although advanced technologies such as single-balloon enteroscopy, DBE, endoscopic abdominal ultrasonography-guided ERCP, and percutaneous transhepatic biliary intervention are the best options for the management of such cases, but they may not be easily available in most developing countries. Laparoscopic exploration of the biliary tract through the afferent or Roux loop of a bilioenteric anastomosis may be a simple and cost-effective method of managing such cases, especially in resource-limited areas of the world.
